# A Two-Locus System Controls Susceptibility to Colitis-Associated Colon Cancer in Mice

**DOI:** 10.18632/oncotarget.177

**Published:** 2010-10-11

**Authors:** Lauren Van Der Kraak, Charles Meunier, Claire Turbide, Serge Jothy, Louis Gaboury, Victoria Marcus, Sing Yun Chang, Nicole Beauchemin, Philippe Gros

**Affiliations:** ^1^ Department of Biochemistry, McGill University, Montreal, Quebec, Canada; ^2^ Goodman Cancer Research Centre, McGill University, Montreal, Quebec, Canada; ^3^ Department of Laboratory Medicine and Pathobiology, St. Michael's Hospital and University of Toronto, Toronto, Ontario, Canada; ^4^ Institut de Recherche en Immunologie et en Cancérologie, Université de Montréal, Montreal, Quebec, Canada; ^5^ Department of Pathology, McGill University Health Centre, Montreal, Quebec, Canada; ^6^ Departments of Medicine and Oncology, McGill University, Montreal, Quebec, Canada

**Keywords:** Colitis-associated colon cancer, genetics, azoxymethane, dextran sulfate, gene mapping

## Abstract

We have previously shown that the differential susceptibility of A/J (susceptible) and C57BL/6J (B6, resistant) mouse strains to azoxymethane (AOM)-induced colorectal cancer (CRC) is controlled by the chromosome 3 locus, *Ccs3*. We report that A/J and B6 mice also show differential susceptibility to colitis-associated colorectal cancer (CA-CRC) induced by combined administration of AOM and dextran sulfate. This differential susceptibility is not controlled by *Ccs3*, but is under distinct genetic control. Linkage analyses in (A/J × B6)F2 mice detected a major CA-CRC susceptibility locus on chromosome 9 (*Ccs4*) which controls tumor multiplicity and tumor surface area. Susceptibility alleles at *Ccs4* are inherited in a recessive fashion, with A/J alleles being associated with susceptibility. We also detected a second locus on chromosome 14 that acts in an additive fashion with *Ccs4*. Strikingly, F2 mice homozygous for A/J alleles at both loci (*Ccs4* and chromosome 14) are as susceptible to CA-CRC as the A/J controls while mice homozygous for B6 alleles are as resistant as the B6 controls, thus supporting the role of two interacting loci in this CA-CRC model. This indicates that susceptibility to chemically-induced CRC and susceptibility to CA-CRC are under distinct genetic control in mice, and probably involve distinct cellular pathways.

## INTRODUCTION

Colorectal cancer (CRC) is the third most common type of cancer-related deaths worldwide [1]. CRC etiologies can be classified as hereditary (10%), sporadic (<90%) or inflammatory (1-2%) [2]. Hereditary CRCs arise due to highly penetrant germline mutations [3]. The two most common syndromes are familial adenomatous polyposis (FAP) and hereditary non-polyposis colon cancer (HNPCC) that arise due to mutations in the *APC* (*Adenomatous polyposis coli*) gene and mismatch repair genes (*MSH2*, *MLH1*, *MSH6* and *PMS2*), respectively. Sporadic CRCs are thought to have a heterogeneous etiology, including a combination of complex environmental and genetic components [4]. Recently, several genome-wide association studies (GWAS) have revealed as many as ten common low-penetrance genetic variants contributing to sporadic CRC risk: these genes or loci are found on human chromosomes 8, 10, 11, 14, 15, 18, 19 and 20 [5-10]. Inflammation, in the form of the inflammatory bowel diseases (IBD), ulcerative colitis (UC) and Crohn’s disease (CD), represents the third most common risk factor for CRC after the FAP and HNPCC [11]. These colitis-associated (CA) CRCs have many similar molecular alterations to sporadic CRCs and therefore were originally considered to be a subtype of sporadic CRCs. However, the timing and frequency of these molecular events differ, which has led to speculations that different genes may be responsible for sporadic CRC and CA-CRC [2, 11]. Both CD and UC are influenced by a variety of environmental and genetic factors including low penetrance susceptibility genes. To date, GWAS studies point to >30 genetic loci/genes for susceptibility to CD and UC, with several shared loci involved in the Th-17 response (*NOD2*, *IL23R*, *IL12B*, *JAK2*, *STAT3*) [12-14]. Allelic differences in the class II human leukocyte antigens (HLA) on chromosome 6 are associated with both increased and decreased risk of CA-CRC [15]. Interestingly, loss of heterozygosity (LOH) on chromosome 6 has been previously reported as a unique feature of CA-CRC, distinguishing it from sporadic CRC and UC [16].

In addition to human GWAS studies, significant contributions have been made towards identifying tumor susceptibility genes using carcinogen-induced mouse models. Sporadic CRC can be modeled through repeated administration of the colon-specific carcinogens 1,2-dimethyhydrazine (DMH) or its metabolite azoxymethane (AOM) [17]. DMH studies in recombinant congenic lines derived from BALB/c (resistant) and STS/A (sensitive) progenitors have identified the *Scc1* locus (*Ptprj* phosphatase) as a major regulator of susceptibility to intestinal tumors [18, 19]. Subsequently, the *Ptprj* phosphatase was shown to undergo loss of heterozygosity (LOH) in sporadic human CRC [20]. Our lab has mapped a novel major CRC susceptibility locus *Ccs3*, on mouse chromosome 3, which controls the differential susceptibility of A/J (susceptible) and C57Bl/6J (B6, resistant) strains to AOM-induced CRC [21].

On the other hand, CA-CRC can be modeled in mice using a single injection of either DMH or AOM followed by intermittent oral administration of inflammation-provoking dextran sulfate (DSS) [22]. This treatment causes thickening of the mucosa, leukocytic infiltration and increased vascular density with permeabilization of the epithelial cell barrier. Several candidate genes have been implicated in CA-CRC in mice. For example, IL-2/b_b2_ and IL-10 appear to play a protective role in CA-CRC, with increased incidence of colonic inflammation and adenocarcinomas in mice bearing inactivating mutations at these genes [23, 24]. Likewise, the key activator NF-κB plays a critical role in inflammation and CA-CRC, and inactivation of the NF-κB activating kinase or TNFα, the upstream activator of NF-κB) results in reduced tumor burden [25, 26]. While the aforementioned genes are crucial in CRC development no studies have been undertaken to identify low penetrance CA-CRC susceptibility loci in mice.

We have undertaken genetic studies in mice to identify genetic loci that regulate susceptibility to CA-CRC, and that may be relevant to human cancer. Here, we show that A/J (susceptible) and B6 (resistant) mice show differential susceptibility to CA-CRC. This is controlled by a novel two-locus system on chromosome 9 (*Ccs4*) and 14 with an additive effect.

## RESULTS

### A/J mice are susceptible to colitis-associated colorectal cancer

**Figure 1:**
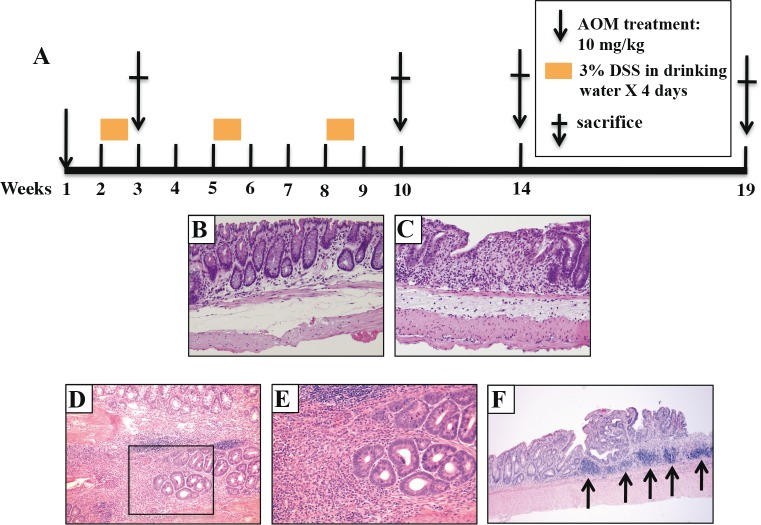
Pathology of AOM/DSS induced tumors in parental A/J and B6 mice (A) Mice were treated with a single AOM injection and 3 consecutive periods of DSS and were sacrificed at either week 3, 10, 14, or 19 post-injection. H&E staining indicates that the animals developed grade 1 (B) or 3 inflammation (C) at 3 weeks (10 × magnification) in A/J and B6 mice, respectively. This is followed by low grade dysplasias at 10 weeks (10 × (D) and 40 × (E) magnification, respectively) and (F) adenomas at 19 weeks (10 × magnification). The arrows indicate sites of grade 3 inflammation in this panel.

In the present study, we investigated whether the two test strains, A/J and B6, differ in response to colitis-associated colon cancer (CA-CRC) development. A/J and B6 mice were treated with a single dose of azoxymethane (AOM) followed by 3 subsequent treatments of dextran sulfate (DSS) (Fig. 1A). Colons were examined at 3, 10, 14 or 19 weeks post-treatment and graded for degree of inflammation, and presence of hyperplastic lesions, and tumors. Inflammation was scored as described in [22]. At 3-weeks post-treatment (Fig. 1B and C) grades 1 and 3 inflammation scores were present in A/J and B6 colons, as defined by either a more scattered lymphocyte infiltration (Fig. 1B) or a larger extended infiltration with increased thickening of the mucosa (Fig. 1C). By 10 weeks post-treatment, B6 mice generally had higher inflammatory scores than A/J mice with low-grade dysplasia becoming apparent in some animals (Fig. 1D and E). At 10 weeks, B6 mice demonstrated larger areas of extended inflammation, as noticed previously [22]. By 19 weeks, flat lesions with clearly defined circular boundaries were identified and scored as hyperplastic lesions. Tubular adenomas were also detected (>0.5 mm diameter) protruding into the lumen of the colon. The multiplicity and surface area of these tumors was used as the second and third quantitative traits in our genetic analyses. These lesions were mainly identified in the distal portion of the colon with scattered tumors being found occasionally in the proximal portion. After 14 weeks, mice showed decreased amounts of inflammation with almost complete disappearance of the inflammation by week 19. On the other hand, A/J mice developed significantly more tubular adenomas relative to their B6 counterparts 14-19 weeks post initiation. An example of such lesions is shown in Fig. 1F after a 19-week treatment period showing grade 3 inflammatory foci (Fig. 1F, arrows). These results indicate that A/J mice, although seemingly less susceptible to initial DSS-induced inflammation, nevertheless are highly susceptible to developing CA-CRC later on, compared to B6 mice.

### CA-CRC susceptibility in A/J is independent of alleles at the *Ccs3* locus

**Figure 2:**
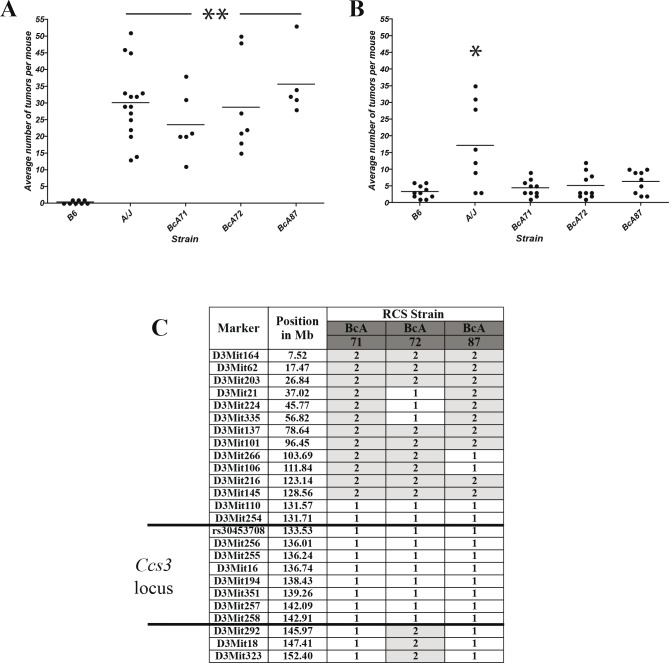
Susceptibility to CA-CRC is not controlled by the *Ccs3* locus Tumor response of parental A/J and B6 mice and RCS strains BcA71, BcA72, and BcA87 mouse strains (encompassing the *Ccs3* locus) to AOM induction (A) or AOM/DSS treatment (B). * *p*<0.01 to B6; ** *p*<0.005 to B6. (C) Haplotype of the above RCS for the *Ccs3* locus. Alleles at each locus are identified as ‘1’ in white (A/J) and ‘2’ in grey (B6). The proximal boundary of the *Ccs3* locus is defined by a resistant strain not shown in this figure.

**Figure 3:**
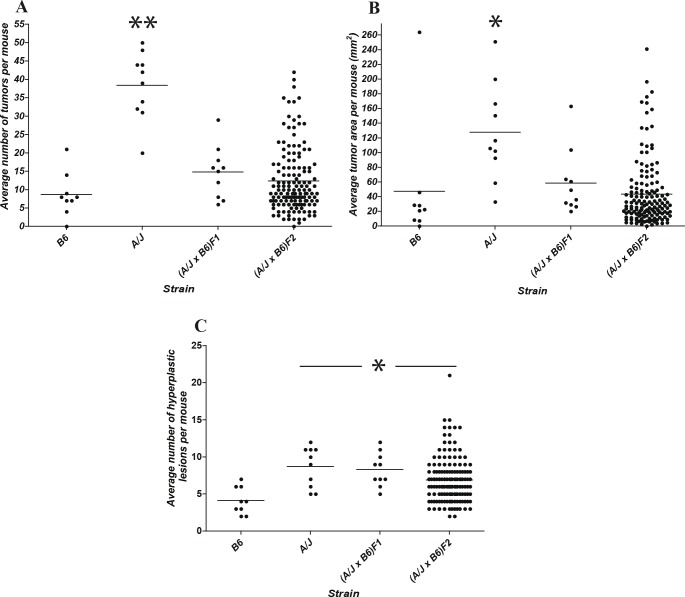
Segregation analysis of susceptibility to CA-CRC induced by the AOM/DSS protocol Distribution of tumor multiplicity (A), tumor surface area (B), and multiplicity of hyperplastic lesions (C) in A/J, B6, (A/J × B6)F1 and 148 segregating (A/J × B6)F2 animals. * *p*<0.05 to B6, ** *p*<0.0001 to B6.

To quantify the differential susceptibility of the A/J and B6 mouse strains to CA-CRC, mice were subjected to the AOM/DSS protocol and their colons examined 19 weeks post-treatment initiation for tumor multiplicity. Results in Fig. 2B show that, on average, A/J mice developed a greater number of colon tumors than their B6 counterpart (*p*<0.01). Although the absolute tumor numbers in the A/J and B6 strains showed experimental variability, we consistently observed significant differences in tumor multiplicity between these two strains in independent experiments (Fig 2B, Fig. 3A).

Inbred A/J and B6 mouse strains are either highly susceptible or fully resistant, to carcinogen-induced CRC, respectively [21]. This is determined by the *Ccs3* locus on chromosome 3. *Ccs3* was mapped as a monogenic trait using phenotype/genotype correlation in a set of AcB/BcA recombinant congenic mouse lines [27]: these mice contain a small amount (12.5%) of DNA from one parent fixed as a set of discrete congenic segments on the background (87.5%) of the other parent. To assess a possible contribution of *Ccs3* to differential susceptibility of A/J and B6 mice to CA-CRC, we phenotyped 3 informative recombinant congenic mouse strains, namely BcA71, BcA72, and BcA87 that carry an A/J derived *Ccs3* chromosomal segment fixed on a B6 background (Fig. 2C). These 3 congenic lines responded to an 8-week AOM induction treatment by developing tumor numbers within the range of those detected in the A/J parents, which were significantly higher than the parental B6 strain (*p*<0.005) (Fig. 2A). However, in the AOM-DSS protocol, the BcA71, BcA72, and BcA87 congenic lines showed low tumor numbers that were similar to those detected in parental B6 controls, and significantly different from those measured in A/J controls (Fig. 2B). These results suggest that the *Ccs3* locus does not impact on the differential susceptibility of A/J and B6 mice to CA-CRC, and that the inter-strain difference for this trait is controlled by other genetic loci.

### Genetic analysis of the CA-CRC susceptibility trait

The mode of inheritance (monogenic *vs* polygenic) of the A/J *vs* B6 differential response to CA-CRC was investigated by segregation analysis in informative (A/J × B6)F1 and F2 mice. (A/J × B6)F1 and F2 mice were treated with the AOM/DSS protocol, and 14 weeks later colons were scored for tumor multiplicity, tumor surface area and number of hyperplastic lesions (Fig. 3A, B and C). As indicated previously, the A/J parental mice displayed a significantly higher number of tumors per mouse (average = 38, *p*<0.0001) and a larger tumor surface area (average = 128 mm^2^, *p*<0.05) relative to the B6 mice (average = 9 for multiplicity, and 47 mm^2^ for surface area). The (A/J × B6)F1 mice showed tumor numbers (average = 14) and surface area (58 mm^2^) that were similar to that of the B6 parent, suggesting that susceptibility to AOM/DSS-induced CRC is inherited as a recessive trait in this cross. Tumor multiplicity and surface area were not normally distributed in the (A/J × B6)F2 animals with a preponderance of resistant animals, suggesting a simple genetic control for a recessive trait associated with susceptibility to CA-CRC. Gender had no significant effect on the phenotypic distribution (data not shown). Since development of CRC is usually preceded by the appearance of hyperplastic lesions, we scored these lesions on the same 14-week treated colons. A/J mice showed approximately a two fold greater number of hyperplastic lesions (average = 9) compared to B6 mice (average = 4), whereas (A/J × B6)F1 and (A/J × B6)F2 mice displayed a similar number of hyperplastic lesions (average = 8) relative to A/J mice, suggesting different patterns of inheritance and possibly different genetic controls for development of tumors *vs* hyperplasic lesions.

### A genome-wide scan reveals a major CA-CRC susceptibility locus on chromosome 9

**Figure 4:**
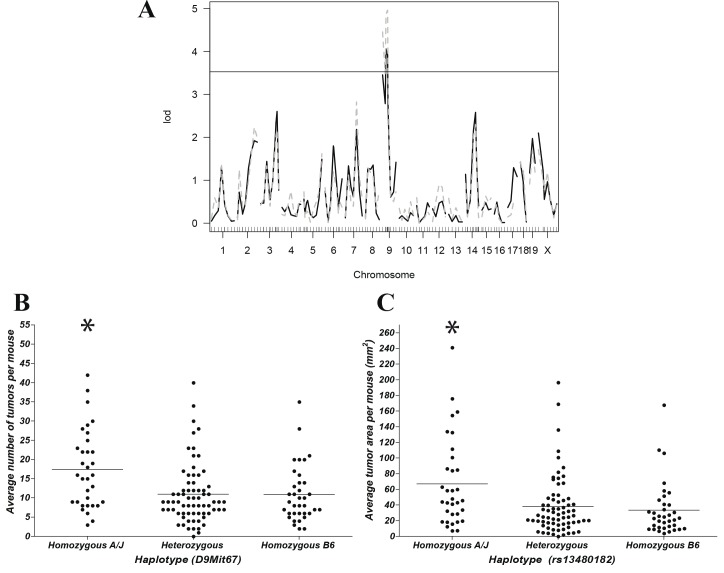
Identification and validation of a chromosome 9 CA-CRC susceptibility locus (A) Genome-wide scan for AOM/DSS-induced tumor multiplicity (black solid line) and AOM/DSS-induced tumor surface area (grey dashed line). The genome scans identified a single significant hit (genome-wide significance threshold is 3.53) on chromosome 9 regulating tumor multiplicity (LOD 4.06) and tumor surface (LOD 4.96) following AOM/DSS treatment. Haplotype association validating the chromosome 9 association at peak markers D9Mit67 with respect to tumor number (B) and rs13480182 with respect to tumor surface (C). * *p*<0.01 to B6.

To further investigate the genetic determinant(s) of CA-CRC susceptibility, a whole genome scan was performed in 148 (A/J × B6)F2 mice using tumor multiplicity and surface area as quantitative traits. In this analysis, genome-wide significance was assessed at a LOD score >3.53. The results presented in Fig. 4A identify one significant linkage on chromosome 9 that influences both tumor multiplicity and tumor area (LOD 4.06 and LOD 4.96, respectively). These results indicate that development of CA-CRC in A/J *vs* B6 mouse strains is controlled by a genetic locus located on chromosome 9. We subsequently assessed the haplotype of the mice at the chromosome 9 peak markers to validate the directionality of the association with respect to tumor number (D9Mit67, 36.8 Mb, Fig. 4B) and tumor area (rs13480182, 49.2 Mb, Fig. 4C). This was congruent with our F1 distribution with mice inheriting two A/J alleles having significantly higher tumor numbers (n = 19, p<0.01) and tumor surface areas (A = 80 mm^2^, p<0.01) than mice inheriting at least one B6 allele. No differences were noted with respect to these measurements in mice inheriting either one or two B6 alleles (n = 11; A = 36 mm^2^ and n = 11; A = 37 mm^2^, respectively). We have attributed this locus the temporary designation *Ccs4*, for colon cancer susceptibility locus 4. *Ccs4* is unrelated to the *Ccs3* locus on chromosome 3 that controls susceptibility to AOM-induced colon tumorigenesis in the same strains, suggesting distinct mechanistic basis for susceptibility to CRC (*Ccs3*) and CA-CRC (*Ccs4*).

### Identification of a chromosome 14 Ccs4 modifier

**Figure 5:**
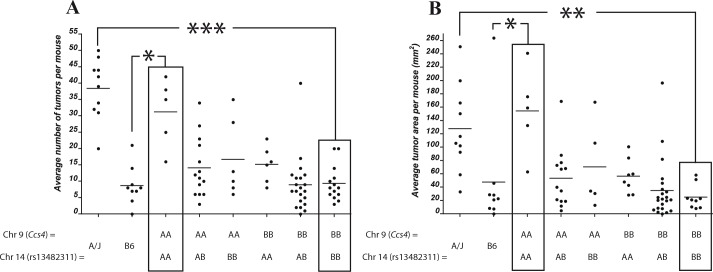
Identification of a chromosome 14 modifier of the *Ccs4* locus Genetic influence of a chromosome 14 locus (peak marker rs13482311) on tumor number (A) or tumor surface (B) for (A/J × B6)F2 mice homozygous for either A/J or B6 alleles at *Ccs4* (D9Mit67 with respect to tumor number and rs13480182 with respect to tumor surface). Black boxes indicate mice that are either homozygous A/J or homozygous B6 across both loci. * p<0.05, ** p < 0.001, *** p < 1 × 10^−6^.

(A/J × B6)F2 mice homozygote for A/J alleles at *Ccs4* are on average more susceptible to CA-CRC with respect to tumor multiplicity and surface area than F2 mice heterozygous or homozygous for B6 alleles at this locus. However, CA-CRC susceptible *Ccs4^a/a^* F2 mice displayed considerable phenotypic variance with respect to tumor numbers and surface area, suggesting the possible contribution of additional genetic loci regulating tumor multiplicity on the permissive *Ccs4a/a* genetic background. This was investigated by a two-dimensional genome scan, followed by assessing the contribution of A/J and B6 haplotypes to tumor numbers for all linkage peaks showing LOD scores >2. Both methods identified a single significant interaction of *Ccs4* with a locus on the distal part of chromosome 14 (peak marker rs13482311, 93.5 Mb). Haplotype analysis showed an additive and very strong effect of the two loci. Two-loci linkage analysis yielded LOD scores of 9.0 and 11.3 for the combined loci, explaining 24.5% and 29.7% of the phenotypic variation for tumor multiplicity and area, respectively. F2 mice that are homozygous for A/J alleles at both chromosome 9 and 14 loci display tumor multiplicity and surface areas comparable to the A/J controls (average = 31 vs 38 and 154 mm^2^ vs 138 mm^2^, respectively) while F2 mice doubly homozygous for B6 alleles at both loci show tumor numbers similar to that of the B6 parental controls (average = 9 vs 8 and 25 mm^2^ vs 47 mm^2^, for multiplicity and surface area, respectively) (Fig. 5). These results identify a two-locus system regulating susceptibility to CA-CRC in mice.

## DISCUSSION

IBD, in the form of UC and CD, represent a major risk factor for CRC. The resulting CA-CRC occurs in a subset of IBD patients, which has prompted the search for low penetrance tumor susceptibility genes, thought to mediate progression of IBD to CA-CRC. Mapping of these genes in humans can be complicated by the vast genetic and environmental heterogeneity of the populations studied and hence inbred mice have been used to study CA-CRC whereby environmental factors and genetic backgrounds can be fixed. Chromosomal regions and individual loci identified by genetic analysis as regulating susceptibility to CA-CRC in mice represent valuable candidates for validation in cohorts of human clinical specimens. We have shown that A/J and B6 mice were susceptible and resistant, respectively to CA-CRC, as modeled using a single injection of AOM and repeated cycles of the inflammatory stimuli DSS. Using an (A/J × B6)F2 cross we mapped a novel two loci system on mouse chromosome 9 (*Ccs4*) and 14, whereby animals homozygous for either A/J or B6 alleles at both loci show susceptibility/resistance characteristics similar to that of the parental strain, indicating that this 2-loci system explain a large portion of the phenotypic variance distinguishing the two parents.

Both sporadic and CA-CRC arise from deregulation of the organized epithelial and stromal cells of the colon and harbor similar molecular alterations underlying CRC development [28]. However, the timing and frequency of these molecular events are different with mutations or LOH at *p53* occurring early in CA-CRC while they arise late in sporadic CRC, and *APC* mutations are rare events in CA-CRC contrary to sporadic CRC [29]. This has led to speculation that appearance and progression of both sporadic CRC and CA-CRC may be influenced by different sets of genes. Results from our study support this contention. Indeed, although A/J is susceptible to both CRC and CA-CRC and although B6 is resistant to both pathologies, results obtained here with subsets of recombinant congenic strains informative for the *Ccs3* locus, together with formal genetic linkage analyses in segregating (A/J × B6)F2s have identified distinct and non-overlapping genetic controls for both traits. Interestingly, the gene for the p105 NF-κB subunit maps within the 5Mb physical interval delineating *Ccs3* and p105-/- mice develop spontaneous intestinal inflammation similar to human IBD [21, 30, data not shown]. It is tempting to speculate that the lack of association of *Ccs3* with CA-CRC in our studies indirectly suggests that it is not an NF-κB-mediated differential response to inflammatory stimulus that is responsible for A/J vs B6 inter-strain differences in susceptibility to CA-CRC.

Scrutiny of published literature and the MGI (Mouse Genome Informatics) database reveal that *Ccs4* overlaps a previously mapped mouse quantitative trait locus (QTL) that regulates response to infection in the form of differential control of tissue repair following injury. A locus regulating differential susceptibility of BALB/c (susceptible) and B6 (resistant) mice to cutaneous leishmaniasis induced by infection with *Leishmania major*, and designated *Lmr2*, has been mapped to a ~10 Mb interval on chromosome 9 that overlaps the genetic interval defined herein for the *Ccs4* locus [31]. The genetic difference at *Lmr2* is expressed as a vigorous wound healing response required for lesion resolution following cutaneous infection with *L. major*. A number of candidate genes have been proposed for *Lmr2* including *Tirap*, *Fli1*, *Il10ra*, as well as members of the *Mmp* family, and *Aplp2*. Recently, a functional promotor polymorphism in the *Fli1* gene has been proposed as a strong candidate for *Lmr2* [31]. The possibility that the *Ccs4*-controlled differential susceptibility to CA-CRC may involve differential response to, and recovery from acute inflammation-induced tissue injury requires further investigation. Indeed, DSS treatment has been shown to cause permeabilization of the epithelial cell barrier resulting in increased cellular turnover to repair tissue damage, a response that may be under the control of *Ccs4*. It is also interesting to note that a similar two-locus system involving chromosome 9 and 14 has been shown to regulate differential brain toxicity of human amyloid precursor protein expressed in transgenic 129sv and FVB mice [32]. Although this two-locus system has been mapped in another strain pair, and with a low degree of resolution, it is nevertheless interesting to note that it regulates response to a toxic substance in the form of different degree to tissue damage.

The *Ccs4* confidence interval is very large and stands at ~ 41Mb delineated by rs29835542/rs3723670 with peak markers D9Mit67 (36.84 Mb) and rs13480182 (49.23 Mb) for tumor multiplicity and area, respectively. The chromosome 14 locus maps to an ~30 Mb region with peak marker rs13482311 (93.5 Mb). The large size of the physical intervals for the two-loci precludes a detailed discussion of positional candidates for the genetic effects on CA-CRC. Nevertheless, using Gene Ontology classification (GO ontology browser) response to stimulus, tissue repair or cancer, several potential candidates arise for the *Ccs4* locus. Interleukin 18 (*IL18*; position 50.4Mb) has been shown to modulate response to chemically-induced colitis in mice [33]. Also, mice bearing a mutation in *CASP12* (53.4Mb), an upstream effector of *IL18*, show increased resistance to acute inflammation, but also increased susceptibility to CA-CRC [34]. Retinoblastoma 1 (*Rb1*; 73.6Mb), a known tumor suppressor, is known to be up-regulated in CRC samples compared to the normal mucosa [35]. The formal identification of the gene responsible for *Ccs4* and associated QTLs on this portion of chromosome 14 will involve the creation and characterization of congenic and sub-congenic lines to narrow down the physical interval of *Ccs4*, as well as the formal evaluation of positional candidates, including the creation and testing of loss-of-function mutations *in vivo*.

The *Ccs4* locus is syntenic to regions found on human Chrs. 11, and 15 while the chromosome 14 locus is syntenic to regions of human chromosome 13. Although more than 30 human IBD loci have been identified with several mapping to the above chromosomes, none overlap with these loci. However, one human CRC susceptibility locus (11q23) falls within the current *Ccs4* interval [6]. The 11q23 locus, associated with a gender-independent increased rectal cancer risk and a moderate colonic cancer risk, maps to a 60 kb region on human chromosome 11 containing 3 ORFs (C11orf53, FLJ45803, LOC120376) and a nearby gene involved in humoral immune response, POU2AF1 [36]. To date no non-synonymous polymorphisms have been identified within this locus and it does not appear to act in cis or trans-regulation with any other known CRC loci.

Potential two-locus systems regulating pre-disposition to colorectal cancer or to inflammatory bowel disease are extremely difficult and statistically challenging to detect in human GWAS studies. The identification of a two-locus system in mice involving chromosome 9 (a possible homolog of the 11q23 human locus) and chromosome 14 provides a novel candidate region of interest and interacting locus which can be readily tested in available GWAS datasets for relevance to related human pathologies.

## MATERIALS AND METHODS

### Ethics Statement

This investigation has been conducted in accordance with the ethical standards and according to the Declaration of Helsinki and according to national and international guidelines and has been approved by the authors’ institutional review board.

### Animals

Inbred A/J, C57BL/6J (B6) and (A/J × B6)F1 mice were originally purchased from Jackson Laboratory (Bar Harbor, ME, USA). The AcB/BcA recombinant congenic strain set was derived from a double backcross (N3) between A/J and B6 parents at McGill University. The breeding scheme used for the derivation of these strains, and genotype data for these animals established for 625 informative markers have been previously described [27]. The (A/J × B6)F2 mice were generated by systematic brother-sister mating from a (A/J × B6)F1 hybrid. All mice were maintained at the Animal Care Facility of McGill University according to the guidelines of the Canadian Council on Animal Care. They were fed regular chow and water ad libitum. All experiments were conducted on mice that were a minimum of 8 weeks of age with a minimum of 5 mice per experimental group. For the duration of the experiments mice were weighed/visually monitored a minimum of twice per week for clinical symptoms. Animals showing signs of discomfort were humanely sacrificed immediately.

### Azoxymethane-Induced CRC

The protocols for induction of colorectal cancer (CRC) by azoxymethane (AOM), the collection of colons, harvesting of tumors and normal mucosa, and scoring methodology were previously described [21]. Briefly, 10 week-old mice were injected with the carcinogen AOM (Sigma, St Louis, MO, USA) once per week for 8 weeks (intra-peritoneal injections of 10 mg/kg). Mice were sacrificed at 19 weeks and the entire colon was fixed in 10% phosphate-buffered formalin and subsequently scored for the number of tumors and hyperplastic lesions.

### Colitis-Associated CRC

Induction of colitis-associated colorectal cancer (CA-CRC) was performed using a combination of AOM and dextran sulfate (DSS Salt Reagent Grade MW 36,000-50,000, MP Biomedicals LCC, Solon, OH, USA). Mice were given a single injection of AOM (10 mg/kg i.p) on day zero, followed by three 4 day cycles of 3% DSS in drinking water. The first treatment was administered exactly a week after the AOM injection with each subsequent treatment 17 days apart. Following the final DSS treatment, mice were given normal water until the end of the experiment (week 14 or 19). The 3% DSS solution was prepared by dissolving DSS in tap water and filter-sterilized using a 0.22m filter (Stericup® Filter Units, Milipore). Fresh 3% DSS was replenished every second day for the duration of treatment. Mice were monitored for DSS consumption during treatment (averaged per cage), with no significant differences being detected amongst the groups. At the end of experiment the mice were sacrificed and their colons collected and tumors enumerated [21]. Tumors were measured using a clear transparency of 1 mm^2^ graph paper and total surface area determined based on the total number of squares overlaying the tumor (measured to the nearest 0.25 mm^2^).

### Histology

The mice were sacrificed and the large intestine (anus to cecum) was removed, washed in PBS and fixed in 10% phosphate-buffered formalin. Fixed tissues were dehydrated in ethanol and paraffin-embedded prior to sectioning (6 μm thickness). Samples were subsequently deparaffinized, mounted onto slides, stained with eosin and counterstained with hematoxylin, following standard histological procedures. Tumor grading and scoring of inflammation according to recommendations of Wirtz et al. [22] was performed by four pathologists (SJ, LG, VM, and SYC). Images were acquired using a Zeiss Axiovert instrument (Zeiss Canada, Toronto, ON, Canada) and processed using Adobe Photoshop (www.adobe.com) [21].

### Linkage and Quantitative Trait Loci (QTL) Mapping

Genomic tail DNA was extracted using a standard proteinase K treatment [27]. The genomic DNA was genotyped for a combined total of 142 single nucleotide polymorphisms (SNPs) and microsatellite markers with coverage at approximately 25 Mb intervals throughout the genome. SNPs and microsatellite markers polymorphic between A/J and B6 were chosen from the Mouse Genome Informatics (http://www.informatics.jax.org/) database. Initial genotyping was performed using a custom-designed panel of SNPs and run using Sequenom iPlex Gold technology. Additional genotyping of microsatellite markers was done by either standard (α-32P)-dATP labeling PCR reactions with separation on 6% denaturing polyacrylamide gels or non-radioactive PCR with subsequent separation on 2%-3% agarose gels and ethidium bromide staining. All QTL linkage analyses were performed using R/qtl and the EM maximum likelihood or Hayley-Knott algorithms [37]. A one-dimensional scan was performed using the scan-one function and empirical genome-wide significance was calculated by permutation testing (1000 tests). To search for possible two gene interactions, a two-dimensional scan was performed using the two-QTL model. Linkage analysis was conducted using tumor multiplicity and tumor surface area as independent phenotypic markers of differential susceptibility to CA-CRC.

### Statistical Analysis

Data is expressed as the mean +/− the standard deviation and all analyses were performed using the Student’s t-test. Results were considered significant if the p ≤ 0.05.
